# Mapping the Homeostatic and Hedonic Brain Responses to Stevia Compared to Caloric Sweeteners and Water: A Double-Blind Randomised Controlled Crossover Trial in Healthy Adults

**DOI:** 10.3390/nu14194172

**Published:** 2022-10-07

**Authors:** Nikoleta S. Stamataki, Shane Mckie, Corey Scott, Douwina Bosscher, Rebecca Elliott, John T. McLaughlin

**Affiliations:** 1 Division of Diabetes, Endocrinology and Gastroenterology, School of Medical Sciences, Faculty of Biology, Medicine, and Health, Manchester Academic Health Science Centre, The University of Manchester, Manchester M13 9PL, UK; 2 Faculty of Biology, Medicine and Health Research and Innovation, Manchester Academic Health Science Centre, The University of Manchester, Manchester M13 9PL, UK; 3 Cargill R&D Center North America, Minneapolis, MN 55447, USA; 4 Cargill R&D Centre Europe, 1800 Vilvoorde, Belgium; 5 Neuroscience and Psychiatry Unit, Division of Neuroscience and Experimental Psychology, School of Biological Sciences, Faculty of Biology, Medicine and Health, Manchester Academic Health Science Centre, The University of Manchester, Manchester M13 9PL, UK; 6 Department of Gastroenterology, Salford Royal Hospitals NHS Foundation Trust, Salford M6 8HD, UK

**Keywords:** stevia, glucose, maltodextrin, food cues, fMRI, physMRI, BOLD, neuroimaging

## Abstract

Non-nutritive sweeteners have potential effects on brain function. We investigated neural correlates of responses to beverages differing in sweetness and calories. Healthy participants completed 4 randomised sessions: water vs. water with stevia, glucose, or maltodextrin. Blood-oxygenation level-dependent (BOLD) contrast was monitored for 30 min post-ingestion by functional Magnetic Resonance Imaging. A food visual probe task at baseline was repeated at 30 min. A significant interaction of taste-by-calories-by-time was demonstrated mainly in motor, frontal, and insula cortices. Consumption of the stevia-sweetened beverage resulted in greater BOLD decrease, especially in the 20–30 min period, compared to other beverages. There was a significant interaction of taste-by-time in BOLD response in gustatory and reward areas; sweet beverages induced greater reduction in BOLD compared to non-sweet. The interaction calories-by-time showed significantly greater incremental area under the curve in thalamic, visual, frontal, and parietal areas for glucose and maltodextrin 10–20 min post-consumption only, compared to water. In the visual cue task, the water demonstrated an increased response in the visual cortex to food images post-consumption; however, no difference was observed for the three sweet/caloric beverages. In conclusion, both sweet taste and calories exert modulatory effects, but stevia showed a more robust and prolonged effect.

## 1. Introduction

Consumption of sugar-sweetened beverages has been associated with an increased risk of weight gain and obesity with higher intakes [1,2]. Non-nutritive sweeteners (NNS) provide sweet taste with minimal or no calories, and could therefore constitute excellent substitutes for caloric sugars, while reducing the available energy and preserving palatability [3]. Recent meta-analyses support a beneficial role of NNS consumption on energy intake and body weight [4,5]. In particular, stevia, a natural zero-calorie containing sweetener, has shown advantageous effects on appetite and energy intake [6,7]. 

Control of food intake involves a complex interaction between homeostatic and hedonic mechanisms, and the human brain plays a central role in this process. It integrates many metabolic, hedonic, and trait-related signals that affect eating behaviour and determine when and how much we eat [8]. The hypothalamus together with the brainstem and the corticolimbic system are regarded as being the core processors in the control of appetite [9]. Caloric sugars and NNS activate functionally connected taste pathways that lead to conscious perception of sweetness, a strong hedonic signal [10,11]. However, they differ in their metabolic fate after ingestion, so that caloric sugars lead to elevated blood glucose, insulin, and satiety inducing gut-peptides postprandially, but NNS consumption has not been shown to affect postprandial metabolism [12,13]. Therefore, it is expected that both overlapping brain regions, derived from the conscious and unconscious sweetness (activation of the gut sweet taste receptors (STRs)), and distinct brain regions due to differences in metabolic consequences and/or their chemical structure, would respond to their consumption. 

Previous studies have shown that glucose ingestion has been associated with a well-established pattern in brain activity, decreased neural activity in the hypothalamus, and the brainstem as measured by blood-oxygenated-level-dependent (BOLD) contrast changes using functional magnetic resonance imaging (fMRI) [14,15,16,17]. Ingestion of NNS has previously been shown to be associated either with no [15] or a transient deactivation of the hypothalamus [18]. Several other areas were associated with decreased BOLD response following glucose intragastric infusion (cerebellum, occipital areas, insula and putamen, parahippocampal, temporal and thalamic regions) [16,19]. To the best of our knowledge, there is no fMRI study investigating the whole brain response to the consumption of a stevia-sweetened beverage.

Satiation attenuates responses in homeostatic and reward-related areas in the brain in response to food tasting and food picture viewing [20]. Under conditions of hypoglycaemia, limbic-striatal brain regions are activated in response to food cues to produce greater desire for high calorie food while following glucose administration responses are attenuated [21]. Food cues (i.e., pictures of food) become more salient under conditions of hunger and less salient under conditions of satiety. Attentional bias to food cues refers to the tendency to focus attention to salient (food) over neutral information. Attention to food cues measured by a reaction time visual dot probe task (VPT) has been previously shown to be higher in the fasted state compared to the fed state [22,23]. This task was used to investigate the neural correlates of food-cue responses during an fMRI investigation. 

In the present study, we aimed to investigate differences in brain activity following oral ingestion of beverages supplying sweetness with calories (glucose), sweetness without calories (stevia), no sweet taste with calories (maltodextrin), or no sweet taste and no calories (water). We used a combination of physiological-fMRI (physMRI) that allowed us to look at BOLD contrast over time following beverage ingestion (signals derived from physiological responses to the consumption of the beverages) compared to a baseline period and fMRI, the examination of neurocognitive responses to food cues 30 min after the consumption of the beverages while performing an attention food related task, the VPT (signals related to hedonics). We hypothesised that glucose consumption will lead to attenuated BOLD in homeostatic and hedonic brain areas in both the physMRI and the task-based fMRI and maltodextrin will show a similar pattern. The consumption of stevia-sweetened beverage was expected to show BOLD responses in overlapping and distinct areas compared to the glucose-beverage.

## 2. Materials and Methods

### 2.1. Participants 

Participants were required to complete 5 study sessions (1 pre-study session and 4 imaging sessions) and were recruited from the University of Manchester (Manchester, UK) and the general Manchester area through advertisements placed around campus and online from November 2019 to December 2020. The study inclusion criteria included healthy men and women aged between 18 and 40 years with body mass index (BMI) within the normal range (18.5–24.9 kg/m^2^), restrained eating score on the Dutch Eating Behaviour Questionnaire ≤ 3, consuming breakfast ≥ 5 times per week, being right-handed, and registered to a General Practitioner in the UK. Exclusion criteria were being diagnosed with a major chronic disease, having intolerances or allergies for products used in the study, weight change ± 5 kg the last 3 months, self-reported anxiety or depression, use of recreational substances within the last month, being pregnant or lactating, self-reported alcohol consumption exceeding 14 units a week, and regular consumption of NNS defined as more than 1 can of diet sodas or more than 1 sachet of NNS per week. Further exclusion criteria related to the Magnetic Resonance Imaging (MRI) were having non-removable metal objects in their body, self-reported claustrophobia, or having had an operation less than 3 months ago. 

The study was approved by the University of Manchester Research Ethics Committee (2019-6814-11707). All participants signed informed consent prior to participation and were compensated for their time. The trial was registered in clinicaltrials.gov under registration NCT04162457. 

Sample size estimation was based on expected BOLD contrast change in the hypothalamus using results of a previous study from our team [24]. The results from the comparison between intragastric saline and 45 g glucose infusion on the hypothalamic BOLD contrast (*n* = 15, peak mean difference of −0.9% change in the BOLD contrast from baseline, and standard deviation of the difference 0.96) were extracted. GPower 3.1 was used to calculate sample size, which calculated that 17 participants are needed for 95% statistical power and an α of 0.05. We recruited a total of 20 participants, out of which 18 completed all imaging sessions. 

### 2.2. Study Design 

This was a randomised, double-blind, crossover study and the participants received four different beverages, one per occasion with at least a 5-day washout period. Both participants and researchers were blinded to the treatments throughout the study and during the analysis. The beverages received identifying codes by the manufacturer (Cargill, Vilvoorde, Belgium), and the codes that matched each beverage were kept in a sealed envelope until after data analysis.

Eighteen participants completed the pre-study session and all four imaging sessions. Participants were asked to have a breakfast of their preference in the morning prior to their scanning sessions, and then fast for 3 or 4 h (no food, only water up to one hour prior to scanning). They were asked to repeat exactly the same breakfast and fasting time prior to each scanning session. Participants received one of the study beverages on each occasion in randomised order, which was also counterbalanced across participants. In detail, randomisation was conducted by an independent person using an online tool (www.random.org accessed on 1 November 2019), creating a randomised and counterbalanced order of study beverages for each participant, which was then followed by the researcher conducting the imaging sessions. 

The study beverages were 330 mL of stevia in water (240 ppm Truvia^®^ Stevia RA95- Rebaudioside A- 95%, Cargill, Vilvoorde, Belgium), 330 mL of 40 g glucose in water, 330 mL of 40 g maltodextrin, or 330 mL water. No additional flavour was added to the beverages. The glucose and stevia beverages were matched for sweetness, the glucose and maltodextrin beverages contained 160 kcal, the water and the stevia beverages contained 0 kcal. The same beverages were administered in a previous study from our group; there was no difference observed in sweetness intensity between glucose and stevia or between water and maltodextrin [6]. 

**Figure 1:**
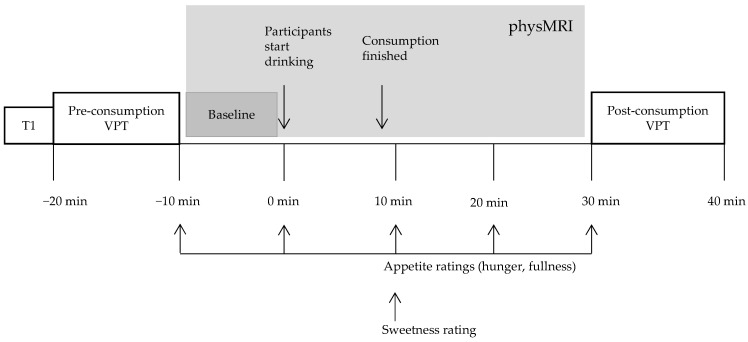
Flow chart of a scanning session. T1, structural scan; physMRI, physiological Magnetic Resonance Imaging; VPT, visual dot probe task. Gray color represents the physMRI part of the imaging.

Beverages were served at room temperature. Drinking was performed in the scanner through an oral silicon tube, lying supine during the fMRI scans. Participants were given 10 min to drink the beverage at a comfortable drinking rate controlled by themselves. The scanning protocol can be found in Figure 1. 

The primary outcomes were the physMRI whole brain responses following the consumption of stevia, glucose, maltodextrin, and water, and the fMRI brain response while performing a food VPT before and after the consumption of the beverages. Secondary outcomes included appetite and sweetness ratings, and the attentional bias to food cues (reaction time). All outcomes are described in detail below.

### 2.3. Pre-Study Session

Participants who were eligible on an online screening questionnaire, which was created to test for the inclusion and exclusion criteria, were invited to a pre-study session at the University of Manchester, Manchester, UK. During this session, we conducted anthropometric measurements and described all details of the study to the participants. In detail, anthropometric measures included body weight measurement by a digital scale in light clothes without shoes (SECA 813 Electronic scale with large platform, Hamburg, Germany), height measured with a portable stadiometer (SECA 213 Portable Height Measure, Hamburg, Germany), and waist and hip circumference (SECA 201 Ergonomic Circumference Measuring Tape, Hamburg, Germany). In addition, during this session participants completed the Dutch Eating Behaviour Questionnaire, and the Three Factor Eating Questionnaire. Participants practiced the fMRI procedure, practiced the VPT, and drinking while lying flat. 

### 2.4. Imaging Sessions

For the MRI sessions, participants arrived between 11:00 a.m. and 2:30 p.m. at the test location (Wellcome Trust Manchester Clinical Research Facility, Manchester, UK) after a fast of 3 or 4 h (no food, only water up to one hour prior to the start of the session). Participants were required to have breakfast of their preference at home, which they repeated before each scanning session, and then fast for 3 or 4 h (fasting time was consistent per participant). Compliance was checked with a breakfast composition questionnaire that participants filled out prior to each scanning session. 

#### 2.4.1. Physiological MRI

This scan followed the pre-consumption VPT scan (see Figure 1). During physMRI participants had to initially undergo a baseline scanning period of approximately 10 min and then were instructed to drink the test beverage for the next 10 min whilst being scanned. Scanning continued for another 20 min after the consumption of the beverage as outlined in Figure 1. 

During the physMRI, participants were asked to indicate their sensation of hunger and fullness on a 10-point scale every 10 min. Subjects had their eyes open and the scales were projected onto a screen visible from inside the scanner. The participant rated each sensation by moving a pointer along the scale, via a response button box held in their right hand. Participants were also asked to rate the sweetness of the beverage after they had consumed it (while in the scanner), and the sensation of thirst before and after the end of a session (in visual analogue scales with pen and paper outside the scanner). 

#### 2.4.2. Visual Dot Probe Task 

Participants performed a VPT twice whilst being scanned, once before the consumption of the test beverage (pre-consumption VPT) and once again 30 min post beverage consumption (post-consumption VPT), as outlined in Figure 1. The VPT involves the presentation of pictures in pairs on screen followed by a dot probe presentation until participant’s response. In the food-related VPT, a picture pair included one food image and one non-food image (stationery). The standardised set of food images from the Full4Health Image Collection was used [25], selected after a preliminary study in house. As a control condition, we included a control VPT where the picture pair consisted of two non-food images (tools and cosmetics). 

A VPT trial begins with the presentation of a fixation cross (1000 ms), then a picture pair (food vs. non food for the food VPT or non-food vs. non-food for the control VPT) appears for another 1000 ms (one at the top and the other at the bottom of the screen). Immediately after the picture pair presentation, a dot probe (a yellow circle on black background) appeared in either the location of the top or the bottom picture and remained for 2000 ms during this time participants were told to respond to the probe by pressing one of the two response keys to indicate dot probe position as quickly and accurately as possible and reaction time was recorded. Each trial was programmed to last exactly 4 s. An attentional bias towards target stimuli (food) exists when there is faster detection of probes replacing such stimuli. 

The VPT task was administered in a block design (Supplementary Materials Figure S1). The blocks were food congruent (the dot appears in place of the food image), food incongruent (the dot appears in place of the non-food image), food mixed (both congruent and incongruent trials), control congruent (the dot appears in place of the cosmetics—the selection of cosmetics as the ‘target category’ in the control task was random), control incongruent (the dot appears in place of the tools), and control mixed. Each block included 8 trials, and each block appeared 3 times in a pseudorandomised order. Total duration of the task was 10 min. The task was presented using Psychopy software (version 1.84.1) [26].

### 2.5. Image Analysis 

#### 2.5.1. MRI Acquisition

Images were acquired with a 3 Tesla Philips Achieva whole-body MR scanner equipped with a standard head coil. The VPT sequence (whole brain T2*-weighted images) was performed twice (before and 30 min after beverage ingestion) using a gradient-echo planar imaging (EPI) (echo time (TE) = 35 ms, repetition time (TR) = 2500 ms, field of view = 240 mm × 240 mm, 44 slices, slice thickness: 3.5 mm, voxel size 3 mm × 3 mm × 3.5 mm). In total, 240 volumes were acquired per run. 

The physMRI sequence (whole brain T2*-weighted images) was performed using EPI and had the following parameters: TE = 35 ms, TR = 2500 ms, field of view = 240 mm × 240 mm, 43 slices, slice thickness: 3.75 mm, voxel size: 3.75 mm × 3.75 mm × 3.75 mm). In total, 960 EPI images were acquired. 

A high-resolution T1-weighted structural image was also acquired for each participant to examine for any structural abnormalities. 

#### 2.5.2. Pre-Processing 

Spatial pre-processing and analysis of imaging data were performed using SPM12 (Wellcome Trust Centre for Neuroimaging, https://www.fil.ion.ucl.ac.uk/spm/software/spm12/ accessed on 1 November 2019), implemented in MATLAB (R2019a, The MathWorks Inc., Natick, MA, USA). Images were firstly realigned using the first image as a reference, then spatially normalised into a standard stereotactic Montreal Neurological Institute (MNI) space using Statistical Parametric Mapping (SPM) templates and then smoothed using a Gaussian kernel filter of 8 mm× 8 mm × 8 mm. 

The Artifact detection Tools (ART) toolbox (http://www.nitrc.org/projects/artifact_detect/ accessed on 1 November 2019) for SPM was used to determine movement artefacts in the scanner. We defined outliers as time points in which framewise global signal deviated more than 3 standard deviations (SDs) from the mean and/or the framewise motion derived from the realignment parameters was greater than 1 mm. Exclusion criteria was more than 15% of outliers in each imaging sequence, including the 10 min physMRI baseline period. On this basis, data from three participants were removed from the physMRI dataset and data from one participant were removed from the task-fMRI dataset. 

#### 2.5.3. physMRI Analysis

Whole brain T2* weighted images were acquired on a 3 Tesla Philips Achieva scanner with single shot, multi-slice echo planar (EPI) pulse sequence. Each volume comprised 34 contiguous axial slices (TR = 2.5 s, TE = 35 ms, 96 × 96 matrix, in-plane voxel size 3.0 mm × 3.0 mm, slice thickness 3.5 mm). A high-resolution T1-weighted structural image was also acquired for each participant for co-registration during pre-processing and to exclude any structural abnormality. Each volume comprised 34 contiguous axial slices (TR = 2.5 s, TE = 35 ms, 96 × 96 matrix, in-plane voxel size 3.0 mm × 3.0 mm, slice thickness 3.5 mm). 

First level analysis was performed using the p-block physMRI analysis technique [27,28], on each subject for each study condition in the following way: the physMRI scans were divided into 20 consecutive 2 min time bins (T01 to T20; T01–T05: baseline, T06–T10: drinking, T11–T20: postprandial), in order to investigate the activation changes over time due to beverage consumption. We did not include in the analysis the T06–T10 time bins due to excessive head movement during that period. The 48 scans from the time bin immediately prior to beverage ingestion (T05) formed the baseline time bin (Tbaseline). In each subject and condition, the signal averages for the 10 post-ingestion time bins (T11–T20) were separately compared to the baseline average (Tbaseline) using regression within the general linear model framework. This resulted in 10 first level images corresponding to the BOLD change from baseline in each successive post-infusion time bin for each subject and condition, which were then used as input to the second level of group-wise analysis. Contrast maps for each time bin were calculated for main effect of taste ((stevia—water) + (glucose—maltodextrin)), main effect of calories ((glucose—stevia) + (maltodextrin—water)), and the interaction taste-by-calories ((stevia—water)—(glucose—maltodextrin)) for each subject. 

To determine whether statistically significant increments in the BOLD signal change from baseline across subjects occurred over time, three repeated-measures ANOVA were conducted, one for the interaction of taste-by-time, one for the interaction of calories-by-time, and one for the interaction of taste-by-calories-by-time. Whole brain analysis was performed and clusters exceeding *p*_FWE_-cluster < 0.05 for cluster extent at a height uncorrected threshold of *p* = 0.001 were considered significant. Beta values were extracted from the significant clusters (mean signal from each cluster) in order to create the time-course graphs that depict the response to each study treatment. 

Moreover, in order to summarise the BOLD over time across the brain for the 3 interactions separately, we applied a more conservative correction for multiple comparisons of peak-level *p*_FWE_ = 0.05. Beta values from a mask including all voxels surviving *p*_FWE_ = 0.05 were extracted in order to create the time course graphs for each beverage. Incremental area under the curve (iAUC) was calculated for each beverage condition and separated into two time bins (10–20 min and 20–30 min). Additional statistical analysis on iAUC using repeated measures ANOVA with beverage type and time as factors was investigated with appropriate post-hoc tests corrected with Bonferroni criterion for multiple comparisons using SPSS (IBM SPSS Statistics Version 23, IBM Corp., Armonk, NY, USA).

#### 2.5.4. Task-Based fMRI Analysis 

For the VPT, we modelled the onset of the VPT stimuli for each beverage condition separately and then created contrasts of interest which were: all food trials > all control trials (both post-consumption), food incongruent trials > food congruent trials (both post-consumption), and the respective post > pre consumption contrasts. Data were high pass filtered at 128 s Then we created contrast images for each predefined contrast of interest for the study treatments comparisons corresponding to the main effect of taste ((stevia—water) + (glucose—maltodextrin)), main effect of calories ((glucose—stevia) + (maltodextrin—water)) and the interaction taste-by-calories ((stevia—water)—(glucose—maltodextrin)). 

In the second level analysis, we performed a one-sample *t*-test with whole brain analysis. As with the physMRI, clusters exceeding *p*_FWE_ -cluster < 0.05 for cluster extent at a height uncorrected threshold of *p* = 0.001 were considered significant. To illustrate the differences between the beverages in areas that showed significant change in the BOLD signal in response to the main effect of taste, calories, and the interaction taste-by-calories, we extracted the mean signal from anatomical masks of the significant clusters for each beverage condition. For illustration purposes and to further follow up significant results, repeated measures ANOVA was performed on the extracted beta values in SPSS (IBM Corp., Armonk, NY, USA).

We also examined the main effect of trial type (food trials > control trials) to investigate whether areas that were expected to activate in response to visual attention to food cues compared to the control task were actually activated independently of the beverage type (whether the paradigm worked). This was conducted via the creation of average contrast images ((water + stevia + glucose + maltodextrin)/4) for the contrasts all food trials > all control trials pre, post and post > pre consumption and then performing one-sample t-tests in SPM. 

The Anatomical Automatic Labelling toolbox (AAL) was used for anatomical labelling of the results.

### 2.6. Statistical Analysis of Behavioural Data 

Non-imaging data were analysed in IBM SPSS Statistics Version 23 (IBM Corp., Armonk, NY, USA). Data are presented as mean ± standard error of the means (SEMs), unless otherwise stated. For the VPT analysis, incorrect responses as well as reaction times (RTs) that were ±3 SDs from the mean were removed. Participants who had >10% incorrect and/or slow responses were excluded. Mean RTs to congruent and incongruent trials (separately for food and control trials) was calculated for each condition and each VPT task (pre- and post-consumption). Attentional bias to food cues was calculated by the following formula: RTmean to food incongruent trials—RTmean to food congruent trials (using all trials from the congruent, incongruent, and mixed blocks). 

Appetite ratings for hunger and fullness were analysed as change from baseline values, AUC was calculated using the trapezoidal rule. These data were analysed using repeated measures ANOVA with beverage type and time (−10, 0, 10, 20, 30 min) as within-subjects variables. Significant interactions revealed by ANOVA were then investigated using post-hoc comparisons and Bonferroni’s correction for multiple comparisons. Not-normally distributed data were analysed with appropriate non-parametric statistics. Specifically, sweetness ratings were not normally distributed; therefore, a Friedman test was conducted, followed by Wilcoxon pairwise tests and Bonferroni correction.

## 3. Results

### 3.1. Participants 

A total of 18 participants completed all 4 imaging sessions. However, due to exclusions described above 15 participants’ data were included in the physMRI analysis and 17 participants’ data were included in the VPT fMRI analysis. A detailed participant flow chart can be found in Supplementary Materials Figure S2. Participants’ characteristics are given in Table 1. Before the start of each imaging session, participants were asked to rate their mood. No significant differences were observed in participants’ mood across the imaging sessions, results are given in Supplementary Materials Table S1. Thirst ratings significantly decreased at the end of each imaging session, with no differences between them.

### 3.2. Appetite and Sweetness Ratings 

**Figure 2:**
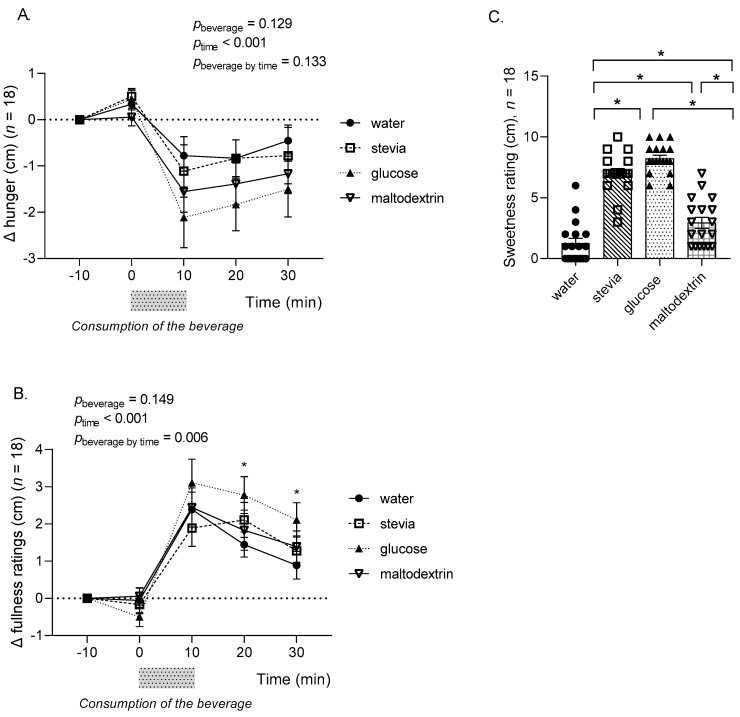
Hunger (**A**), fullness (**B**), and sweetness ratings (**C**) following the consumption of water, stevia, glucose, and maltodextrin beverages. * *p* < 0.05 (in panel B the asterisk indicates *p* < 0.05 between water and glucose). ∆, change.

A repeated measures ANOVA with beverage type (water, stevia, glucose, maltodextrin) and time (−10, 0, 10, 20, 30 min) as within-subjects variables was conducted for hunger ratings (change from baseline values) and revealed a main effect of time (F(2, 27) = 12.94, *p* < 0.001, Greenhouse–Geisser). However, no effect of beverage type (*p* = 0.129) or interaction between beverage type and time (*p* = 0.133) (Figure 2A,B) was demonstrated. Similar analysis was conducted for the fullness ratings and revealed a significant main effect of time (F(2, 25) = 26.20, *p* < 0.001, Greenhouse–Geisser) and a significant interaction between beverage type and time (F(6, 94) = 3.39, *p* = 0.006, Greenhouse–Geisser). Post hoc tests revealed a significant increase in fullness ratings at 20 and 30 min following the consumption of the glucose beverage compared to water beverage (*p* = 0.016 and *p* = 0.047 at 20 and 30 min, respectively); however, there was no difference in fullness between the stevia, glucose, and maltodextrin beverages (all *p* > 0.05). 

Participants were asked to rate the sweetness of the beverage they consumed immediately after the end of the drinking period (10 min). Results are given in Figure 2C and showed that the glucose and stevia beverages were perceived as significantly sweeter compared to the water and maltodextrin beverages (all *p* < 0.001), and maltodextrin slightly but significantly sweeter than water (*p* = 0.02). There was no significant difference in perceived sweetness between the glucose and the stevia beverages in line with the design of the study. 

### 3.3. Visual Probe Task

A 4 × 2 × 2 repeated measures ANOVA was conducted with beverage type, time (pre- and post-beverage consumption), and congruency (congruent, incongruent trials) as within-subjects variable and reaction time as the dependent variable. There was no significant main effect of beverage type, time (pre and post beverage consumption), congruency or a significant interaction. We calculated attentional bias to food cues for each beverage condition pre- and post-consumption, no significant differences were observed. Similar analyses were conducted for the control condition. Results showed that there were no significant differences in the control trials (cosmetics vs. tools).

### 3.4. Neuroimaging Results 

#### 3.4.1. physMRI

- Areas where BOLD signal changed in response to the interaction of taste-by-calories-by-time

Results of the one-way ANOVA investigating the differences in BOLD signal responses over time in response to the interaction of taste-by-calories-by-time are presented in Table 2. The cluster extent of 5 clusters was significant. These clusters were observed in the precentral and postcentral gyrus, supplemental motor area, middle cingulate gyrus, left middle/superior frontal gyrus, insula, and left transverse temporal gyrus. The average time courses for the significant clusters are presented in Supplementary Materials Figure S3. 

**Figure 3:**
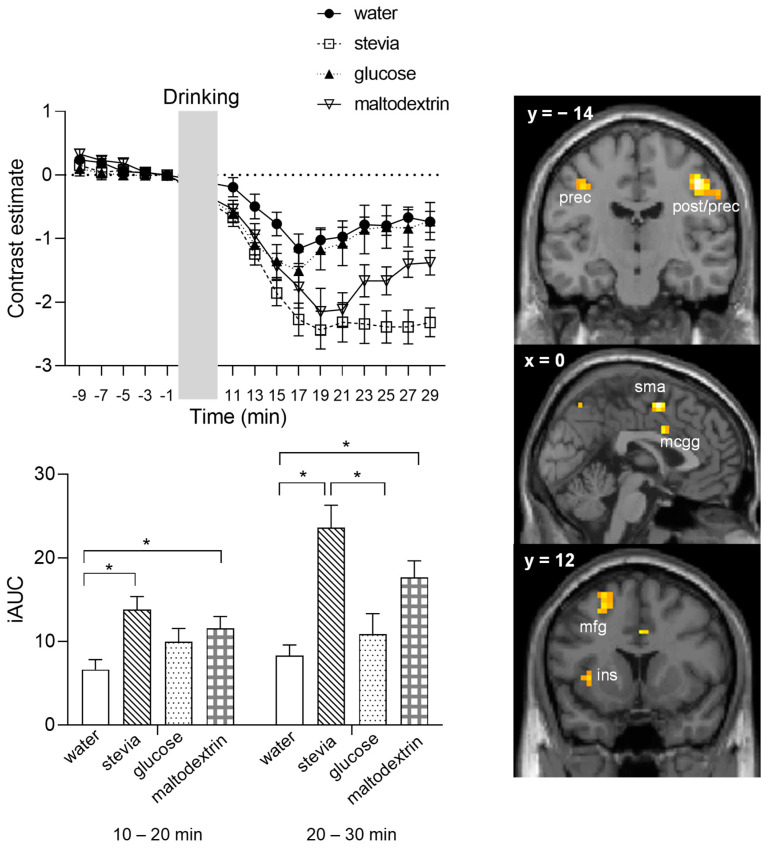
Mean blood-oxygenation level-dependent (BOLD) signal response for the interaction of taste-by-calories-by-time across all voxels that survived a peak-level corrected for multiple comparisons threshold of *p*_FWE_ < 0.05. Brain sections show significant activations from the whole brain analysis (*p*_FWE_ < 0.05 corrected), bar graph shows the incremental area under the curve separated in two time bins, 10–20 min and 20–30 min after beverage consumption. * *p* < 0.05, applying Bonferroni correction. ins, insula; mcgg, middle cingulate gyrus; mfg, middle frontal gyrus; post, postcentral gyrus; prec, precentral gyrus; sma, supplementary motor area; iUAC, incremental area under the curve.

Figure 3 demonstrates the time-course graph of the mean beta values from all activated voxels in response to the interaction taste-by-calories-by-time. In the first 10–20 min time bin, iAUC for stevia and maltodextrin is significantly higher compared to water. In the second time bin, 20–30 min, the differences are maintained, and the iAUC for stevia is also significantly different to glucose, with stevia showing a persistent BOLD signal response. Calculation of the iAUC for the 25–30 min postprandial period shows that stevia iAUC is also marginally significantly different to maltodextrin as well (*p* = 0.06).

- Areas where BOLD signal changed in response to the interaction taste-by-time

Results of the one-way ANOVA investigating the differences in BOLD signal responses over time following the ingestion of the sweet beverages (stevia, glucose) relative to the non-sweet beverages (water, maltodextrin) are presented in Table 2. The cluster extent of 5 clusters was significant. The clusters were observed in the right putamen, superior and middle frontal gyrus, insula, inferior frontal gyrus, anterior and middle cingulate cortex, right supramarginal gyrus and inferior parietal lobule, and right fusiform/hippocampus. In these clusters, the BOLD signal response following stevia and glucose was significantly lower compared to water and maltodextrin. Time-course graphs showing the BOLD response over time for each beverage are presented in Supplementary Materials Figure S4.

**Figure 4:**
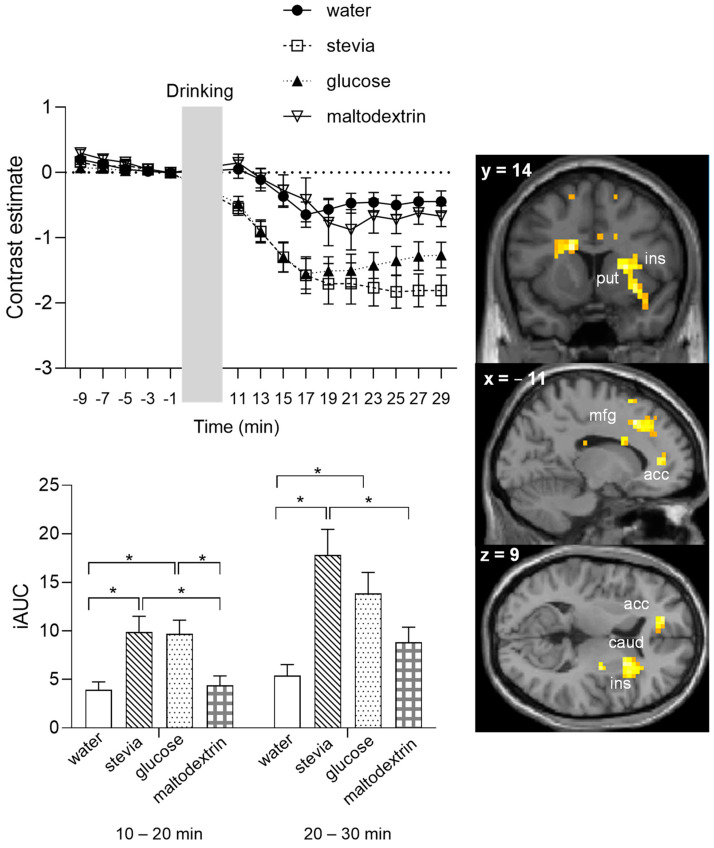
Mean BOLD signal response for the interaction taste-by-time across all voxels that survived a peak-level correction for multiple comparisons at a threshold of *p*_FWE_ < 0.05. Brain sections show significant activations from the whole brain analysis (*p*_FWE_ < 0.05 corrected), bar graph shows the incremental area under the curve separated in two time bins, 10–20 min and 20–30 min after beverage consumption. * *p* < 0.05, applying Bonferroni correction. acc, anterior cingulate cortex; mfg, medial superior frontal gyrus; caud, caudate; ins, insula; put, putamen.

Figure 4 presents the time-course graph of the mean beta values from all activated voxels in response to the interaction of taste-by-time, which survived a more conservative peak threshold corrected for multiple comparisons of *p*_FWE_ = 0.05. Calculation of the iAUC in two time bins, 10–20 min and 20–30 min post-consumption, showed that the effect of taste was apparent in the 10–20 min time bin, with stevia and glucose showing a higher iAUC compared to water and maltodextrin. In the 20–30 min post-consumption, stevia maintains the difference from water and maltodextrin, but glucose does not (glucose iAUC is significantly different to water but not to maltodextrin), suggesting the possibility of a more persistent reduction of BOLD signal after the stevia beverage.

- Areas where BOLD signal changed in response to the interaction calories-by-time

Results of the one-way ANOVA investigating the differences in BOLD contrast responses over time following the ingestion of the caloric (glucose, maltodextrin) relative to the non-caloric beverages (water, stevia) are presented in Table 2. The cluster extent of 9 clusters was significant. These clusters were observed in the thalamus, calcarine cortex, lingual gyrus, precuneus, cerebellum, right inferior and superior frontal gyrus, right rolandic operculum/insula, left postcentral, left putamen, left hippocampus, supplemental motor area, and left angular gyrus. The average time courses for each significant cluster are presented in Supplementary Materials Figure S5. 

**Figure 5:**
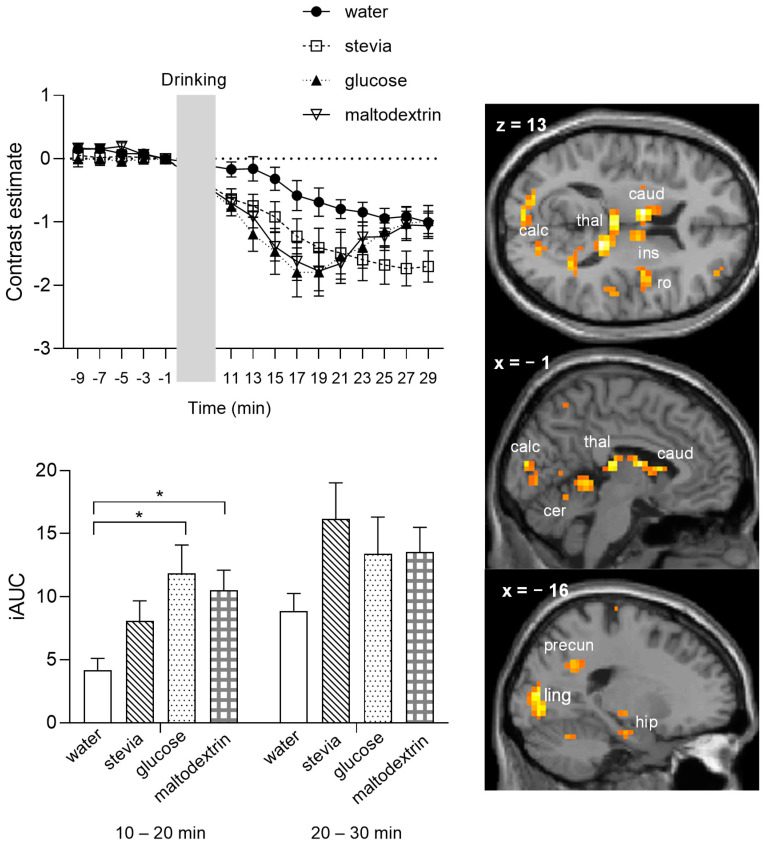
Mean BOLD signal response for the interaction calories-by-time across all voxels that survived a peak-level corrected for multiple comparisons threshold of *p*_FWE_ < 0.05. Brain sections show significant activations from the whole brain analysis (*p*_FWE_ < 0.05 corrected), bar graph shows the incremental area under the curve separated in two time bins, 10–20 min and 20–30 min after beverage consumption. * *p* < 0.05, applying Bonferroni correction. Calc, calcarine cortex; caud, caudate; cer, cerebellum; hip, hippocampus; ins, insula; ling; lingual gyrus; precun, precuneus; ro, rolandic operculum; thal, thalamus.

Figure 5 presents the time-course graph of the mean beta values from all activated voxels in response to the interaction calories by time, which survived a more conservative peak threshold corrected for multiple comparisons of *p*_FWE_ = 0.05. In the first 10–20 time bin, iAUC for glucose and maltodextrin was significantly higher compared to water, but there was no difference between stevia and the other beverages. In the 20–30 min time bin, there was no longer a significant difference between the water and the caloric beverages, since the BOLD signal in the glucose and maltodextrin conditions tended to return to preprandial values as illustrated in the time course graph. Moreover, in the last 5 min time bin (25–30 min), the iAUC was significantly different between water and stevia.

#### 3.4.2. Task-Based fMRI 

We investigated the effect of beverages consumption differing in taste (sweet or not sweet) and caloric content (with or without calories) on the brain’s response to a food visual dot probe task and a control visual probe task before and 30 min after ingestion in healthy normal-weight participants. The contrasts of interest were: all food trials > all control trials, food incongruent trials > food congruent trials, post > pre (all food trials > all control trials), and post > pre (food incongruent trials > food congruent trials). 

**Figure 6:**
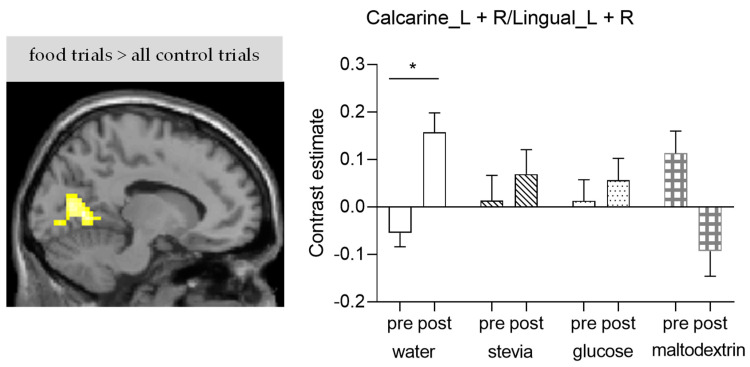
Significant differential brain activation during the visual dot probe task compared to the control task post versus pre-consumption, in response to the interaction between taste and calories. Brain sections show significant clusters from the whole brain analysis (*p* < 0.001, uncorrected), bar graph shows the average contrast estimate in arbitrary units (±standard error of the mean (SEM)) at the significant cluster (*n* = 17); * *p* < 0.05, Bonferroni correction applied.

Results from the whole brain analysis of the VPT are summarised in Table 3. We observed a statistically significant differential BOLD response in a cluster including the calcarine cortex and lingual gyrus bilaterally in response to the taste-by-calories interaction for the contrast post > pre (all food trials > all control trials) (Figure 6). In particular, after the consumption of water BOLD signal was significantly increased in this cluster; however, activity did not change significantly after consumption of the stevia, glucose, and maltodextrin beverages. We did not observe any other significant differences in brain activation in response to the main effect of taste or calories for any of the contrasts of interest. 

We examined the main effect of trial type (all food trials > all control trials) to investigate whether areas that were expected to activate in response to visual attention to food cues were actually activated (Table 3). Results showed that the pre-consumption state activation in response to food trials compared to control trials increased in a cluster including the left frontal inferior gyrus and decreased in a cluster encompassing part of the right fusiform and lingual gyrus. In the post-beverage consumption state brain activity increased in the caudate, thalamus, superior frontal gyrus, angular gyrus, parietal inferior lobule (all bilaterally) and left amygdala and hippocampus in response to food trials compared to control trials.

## 4. Discussion

We have demonstrated for the first time the whole brain response following the ingestion of a stevia-sweetened beverage along with controls for sweet taste and calories. We used a combination of physMRI and task-based fMRI to examine (i) the brain signals derived solely from the ingestion and the subsequent physiological responses associated with beverage consumption and (ii) food-cue responses before and after beverages within the same study, in an attempt to examine both homeostatic and hedonic signals associated with sweet beverage consumption. 

In summary, this study showed a significant interaction of taste-by-calories-by-time mainly in motor, frontal areas, and insula; in these clusters, consumption of the stevia-sweetened beverage resulted in greater BOLD contrast decrease especially in the 20–30 min post-consumption period compared to the consumption of the other beverages. Moreover, sweet beverage consumption was associated with a greater attenuation of activity over time in areas involved in taste and reward processing compared to non-sweet beverages. In areas responding to caloric compared to non-caloric beverages over time including thalamic, visual, parietal, and frontal areas, among others, glucose and maltodextrin demonstrated a significant decrease in brain activity until 20 min after the consumption only compared to water, stevia showed a delayed and longer-lasting BOLD decrease. In the food-cue task, the consumption of the water demonstrated an increased response in the visual cortex to food images; however, no difference in the BOLD contrast was observed following the consumption of the three sweet/caloric beverages (stevia, glucose, maltodextrin). 

The comparison of BOLD contrast response to taste-by-calories-by-time led to less extensive activation compared to the other comparisons and involved mainly the primary somatosensory cortex (postcentral gyrus) and primary motor cortex (precentral gyrus), supplemental motor area, cingulate gyrus, middle/superior frontal gyrus, and insula. From the average BOLD contrast time course graph and the iAUC, we concluded that the interaction in the first 10 min post consumption (10–20 min) was driven by stevia and maltodextrin eliciting a significant reduction in BOLD signal response over time compared to water, while in the 20–30 min, stevia also showed a significant BOLD signal decrease compared to glucose (and marginally different to maltodextrin in the last 25–30 min time bin). Overall and in almost all interactions studied, the consumption of the stevia beverage induced a slower and more gradual reduction in BOLD signal response compared to the pre-consumption baseline, which remained until at least 30 min post-ingestion. Therefore, the possibility of stevia having a specific effect in the brain cannot be excluded and may be attributed to its metabolic fate after ingestion. In vivo studies in animal models have shown that steviol glycosides are not metabolised in the upper gastrointestinal tract but are degraded slowly in the lower gastrointestinal tract by colonic bacteria, leading to a long slow increase in portal and plasma levels of steviol or its metabolite [29]. Steviol detection in portal plasma has been demonstrated to sustain over a period of hours [30]. Future research could examine the brain response to the consumption of stevia beyond the 30 min period to investigate when the signal returns to baseline and the use of intragastric infusion would help to isolate the gut-to-brain signalling induced by stevia consumption. 

We demonstrated significant differential BOLD responses for sweet versus non-sweet beverages over time. The areas of the brain where BOLD signal was reduced in response to sweet beverage ingestion over time included areas of the corticolimbic system associated with reward (prefrontal cortex, putamen, caudate) and gustatory-related areas with the main representative being the insula and cingulate cortex. Previous fMRI studies have demonstrated similar brain activation patterns during caloric and non-caloric sweeteners tasting in the primary taste processing areas (anterior insula, frontal operculum) but differential brain activity in reward-related areas (striatum, midbrain), which responded to caloric but not non-caloric sweeteners tasting, proposing that low-calorie sweeteners might be less rewarding [31,32]. However, a recently published systematic review reported that commonly activated areas between caloric and non-caloric sweeteners are the insula/operculum, cingulate, and the striatum and homeostatic areas (hypothalamus, brainstem) [33]. It is important to note that the above studies have not examined the BOLD response over time, but only the immediate effect of tasting. Our results show deactivation of the gustatory and reward areas by both glucose and stevia in the 30 min postprandial period, a long-lasting signal occurring potentially beyond oral sweet tasting. 

In the comparison of caloric versus non-caloric beverages over time, the significant clusters demonstrated the engagement of large visual, motor and parietal areas, thalamus, cerebellum, insula, hippocampus, and prefrontal cortex. A previous fMRI study demonstrated that intragastric glucose infusion is associated with reduced BOLD contrast response in the cerebellum, right fusiform, and lingual gyri, insula and putamen, left parahippocampal gyrus, temporal and thalamic regions, most of which overlap with our results in this comparison (caloric versus non-caloric beverages) [16]. From the average BOLD signal response in all activated areas for the interaction calories-by-time, it is evident that the caloric beverages induce a BOLD signal decrease only in the 10–20 min post consumption and only compared to water as illustrated by the iAUC. After that point, BOLD signal tends to return to preprandial values by 30 min post consumption and the difference to water is no longer significant. Stevia BOLD response in these areas did not differ to either water or caloric beverages, but a slower and more delayed BOLD decrease was noted. The pattern of the response could be in line with the time course of calories from the beverages being processed, absorbed into bloodstream, and when glucose is no longer in excess BOLD returns to pre-prandial levels. The peak decrease in BOLD contrast was demonstrated between 18 and 20 min post-consumption. Glucose and maltodextrin lead to similar increases in blood glucose and insulin concentrations according to previous research [15]; in a previous study from our group, blood glucose response to glucose and maltodextrin was still significantly increased compared to water and stevia at 30 min postprandially [6]. 

The consumption of water compared to all other beverages in this study (stevia, glucose, maltodextrin) led to significant increased activation in response to food trials (food versus non-food images) compared to control trials (non-food versus non-food images) in a cluster encompassing part of the visual cortex, including the calcarine cortex and lingual gyrus bilaterally. Even though the visual cortex is not considered a direct modulator of appetitive responses, processing of visual stimuli is highly dependent on motivational factors. Visual cortex activation is apparent in studies that use visual cues to induce craving [34] and has been also associated with the motivational salience of food cues (i.e., high versus low calorie food cues) [35]. Previous research has shown that there is strong modulation of the visual cortex by food cues even immediately after glucose ingestion [36] and up to 120 min postprandially [37], proposing that activation of visual cortex is also dependent on metabolic signals. Our results further confirm this finding, the increased BOLD in the visual areas observed after water consumption was not observed following the consumption of the caloric beverages (glucose, maltodextrin) and also the stevia beverage (providing only sweet taste). A recent fMRI study has also shown similar results following sucralose ingestion during a food decision task. Sucralose versus water led to decreased activation in a range of areas including the visual cortex [38]. Given that visual cortex responds to metabolic state differences in response to food cues and that higher-value targets induce greater visual activation [39], we could hypothesise that altered salience of the food cues mediated the effect in the visual cortex. This could be interpreted as food cues being less salient after the consumption of the sweet (stevia), caloric (maltodextrin) or sweet and caloric (glucose) beverages compared to water. 

Among the limitations of this study is that we did not measure any metabolic markers such as blood glucose, insulin, or gut peptide concentrations. This was intentional in the design process due to the technical difficulties and disruptive nature of blood collection during an fMRI investigation, which in turn could also introduce a lot of noise in the acquired images. Another limitation is that for the physMRI, we finally included the results of 15 participants, even though the power calculation indicated a required sample size of 17; results should be interpreted with caution. Participants were inevitably placed in supine position and this could have influenced gastric emptying and post-prandial metabolic responses, and also the timing of the BOLD change that is sensitive to metabolic signal changes. However, we conducted a preliminary pilot study to ensure that blood glucose time-course is different between nutritive and non-nutritive sweeteners while participants are lying flat when consuming the beverage and remain in this position for the duration of the scan. Including the oral phase of ingestion in the physMRI has both advantages and disadvantages, but in our design, taste was a key variable. Inclusion of the oral phase allows for cephalic and cognitive factors to occur, and most closely reflects actual ingestion of sweeteners. On the other hand, including the oral phase adds head movement due to swallowing, which was the main reason why we excluded the drinking part in the physMRI analysis. 

Despite their widespread use, we are only beginning to understand the effects of NNS consumption response in the brain. Future work should focus on the potentially differential effects among different NNS type consumption on brain responses, as they have already been demonstrated to exert differential effects on body weight [40]. Another other important avenue for future work will be to dissect the pure gut-to-brain signalling following the stevia beverage compared to appropriate controls. Direct infusion into the gut will shed light into which of the activations were due to the precedent oral sweet and which were derived solely from gut-derived signals. 

In summary, this study demonstrated attenuation of the brain response to both caloric and sweet beverages consumption, with stevia showing a more prolonged effect. All other beverages in this study demonstrated attenuated brain activity to food cues compared to water in the visual cortex post consumption. It seems unlikely that the brain response after stevia is solely driven by the brief event of sweet tasting in the mouth; other neurophysiological effects such as its transport and metabolic fate may be involved and could potentially be linked to effects on feeding behaviour.

## Figures and Tables

**Table 1:** Subjects’ characteristics.

	*n* = 18
Age (years)	26 ± 5
Weight (kg)	60.1 ± 11.8
Body mass index (kg/m^2^)	21.5 ± 2.1
Height (cm)	166 ± 9
Waist circumference (cm)	71.6 ± 7.2
Hip circumference (cm)	95.3 ± 9.9
Dutch Eating Behaviour Questionnaire	
Restrained	1.7 ± 0.5
Emotional	2.0 ± 0.6
External	2.9 ± 0.6
Three Factor Eating Questionnaire	
Cognitive restraint	3.7 ± 2.4
Disinhibition	3.4 ± 2.0
Hunger	3.8 ± 2.3

Values are means ± standard deviations (SDs).

**Table 2:** Significant clusters exhibiting interactions of interest at *p* < 0.001 (uncorrected), *n* = 15.

Size at *p* < 0.001	*p*_FWE-C_	F	Region	MNI Coordinates		
				x	y	z
Taste-by-Time Interaction						
1039	<0.001	8.12	Putamen_R	26	−11	13
		7.63	Frontal_Sup_L + R	−12	19	43
		7.38	Frontal_Sup_Medial_L	−8	27	39
		7.07	Insula_R	37	19	−14
		5.38	Cingulum_Mid_R + L	14	−18	39
113	<0.001	6.89	ACC_pre_L/ Frontal_Sup_L	−16	46	9
39	0.010	5.44	Insula_L	−35	19	−10
		3.54	Frontal_Inf_Orb_L	−27	16	−18
114	<0.001	5.28	Supramarginal_R/Parietal_Inf_R	52	−33	24
54	0.002	5.09	Hippocampus_R/Fusiform_R	33	−33	−6
Calories-by-time interaction						
1604	<0.001	10.50	Thalamus_L + R	−8	−3	13
		10.10	Lingual_L + R	29	−56	−6
		9.17	Calcarine_L + R	−16	−82	9
		7.01	Cerebellum_L + R	7	−45	−3
217	<0.001	6.35	Frontal_Inf_Oper/Tri_R	22	−15	31
		6.22	Insula_R/ Rolandic_Oper_R	37	−3	13
56	0.001	6.27	Postcentral_L	−1	16	46
40	0.008	6.03	Hippocampus_L/Parahippocampal_L	−1	−60	58
30	0.027	5.99	Frontal_Sup_R	33	53	9
34	0.017	5.46	Suppl_Motor_Area_L + R	−1	16	46
38	0.010	5.46	Precuneus_L + R	−1	−60	58
75	<0.001	5.14	Angular_L/Parietal_Inf_L	−35	−67	39
50	0.003	4.85	Putamen_L	−27	−3	−10
Taste-by-calories-by time interaction						
137	<0.001	8.63	Postcentral_R/Precentral_R	44	−15	46
305	<0.001	8.04	Suppl_Motor_Area_L + R	−1	1	54
		7.91	Cingulum_Mid_L + R	−12	−7	35
		5.40	Frontal_Sup/Mid_L	−23	12	54
39	0.006	5.70	Heschl_L	−21	−26	5
100	<0.001	5.69	Postcentral_L/Precentral_L	−38	−11	43
24	0.044	5.12	Insula_L	−35	12	1

Regions were defined using the automatic anatomical labelling. ACC, anterior cingulate cortex; MNI, Montreal Neurological Institute.

**Table 3:** Regions demonstrating significant difference in brain activation in response to food trials vs. controls trials pre- and post-beverage ingestion in healthy participants, *n* = 17.

Size at *p* < 0.001	*p*_FWE -C_	z	Region	MNI Coordinates		
				x	y	z
Interaction Taste-by-Calories Post- > Pre-Consumption (All Food Trials > All Control Trials)						
184	<0.001	4.03	Calcarine_L + R/Lingual_L + R	−9	−64	4
Main effect of trial type: food trials > control trials Pre beverage consumption						
74	0.035	3.79	Frontal_Inf_Oper_L/ Frontal_Inf_Tri_L	−48	14	18
114	0.007	4.86	Fusiform_R/Lingual_R	27	−46	−14
Main effect of trial type: food trials > control trials Post-beverage consumption						
321	<0.001	4.97	Caudate_L + R, Thalamus_L + R, Putamen_L	321	21	38
162	0.001	4.79	Frontal_Sup_L + R	162	15	41
205	<0.001	4.67	Angular_L	205	−36	−52
130	0.002	4.08	Angular_R	130	42	−55
135	0.002	4.55	Amygdala_L, Hippocampus_L	135	−18	−4
72	0.031	4.36	Temporal_Inf_L	72	−48	−64
80	0.021	3.92	Frontal_Mid_R	80	27	11

Threshold set at *p* < 0.001 uncorrected (cluster-level). MNI, Montreal Neurological Institute.

## Data Availability

Data available upon request to the corresponding author.

## Supplementary Materials

The following supporting information can be downloaded at: https://www.mdpi.com/article/10.3390/nu14194172/s1, Figure S1: Illustration of the visual probe task. Example of a (A) food congruent trial, (B) food incongruent trial, and (C) block design of the visual probe task; Figure S2: Participant flow chart. Figure S3: Line graphs present changes in blood-oxygenation-level-dependent (BOLD) signal over time in clusters that showed a significant interaction of taste-by-calories-by-time, *n* = 15; Figure S4: Line graphs present changes in blood-oxygenation-level-dependent (BOLD) signal over time in the significant clusters following oral ingestion of the sweet (stevia, glucose) compared to the non-sweet beverages (water, maltodextrin), *n* = 15; Figure S5: Line graphs present changes in blood-oxygenation-level-dependent (BOLD) signal over time in selected clusters that showed a significant effect of time in the comparison of caloric (glucose, maltodextrin) compared to the non-caloric beverages (water, stevia), *n* = 15. Table S1: Participants’ mood rating before the start of each imaging session, *n* = 18. *p* values correspond to repeated-measures ANOVA with beverage type as within-subjects variable.
