# Supplementary material for: Mapping the Homeostatic and Hedonic Brain Responses to Stevia Compared to Caloric Sweeteners and Water: A Double-Blind Randomised Controlled Crossover Trial in Healthy Adults
Source: Nutrients. 2022 Oct 7;14(19):4172. doi: 10.3390/nu14194172 (PMC9570671; doi:10.3390/nu14194172)
Supplement: Supplementary file 1 [file nutrients-14-04172-s001.zip › nutrients-1933689-supplementary.pdf]

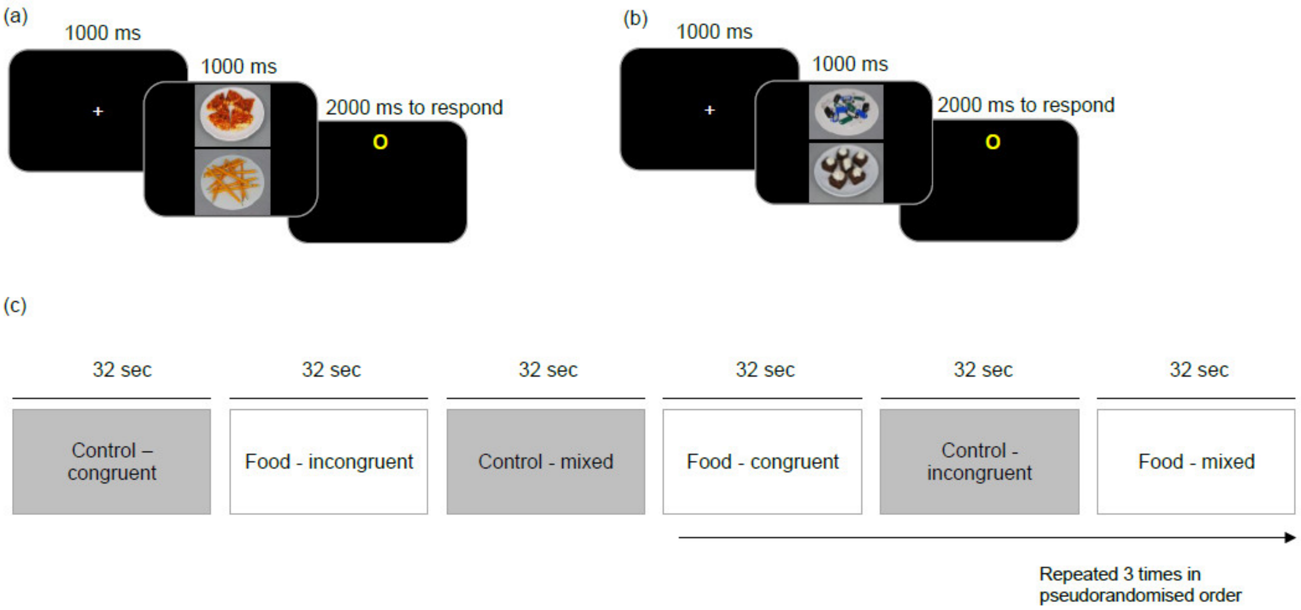

Figure S1. Illustration of the visual probe task. Example of a (**a**) food congruent trial, (**b**) food incongruent trial and (**c**) block design of the visual probe task. In a food congruent trial the dot appears in the position of the food image, while in an incongruent trial the dot appears in the position of the non-food image.

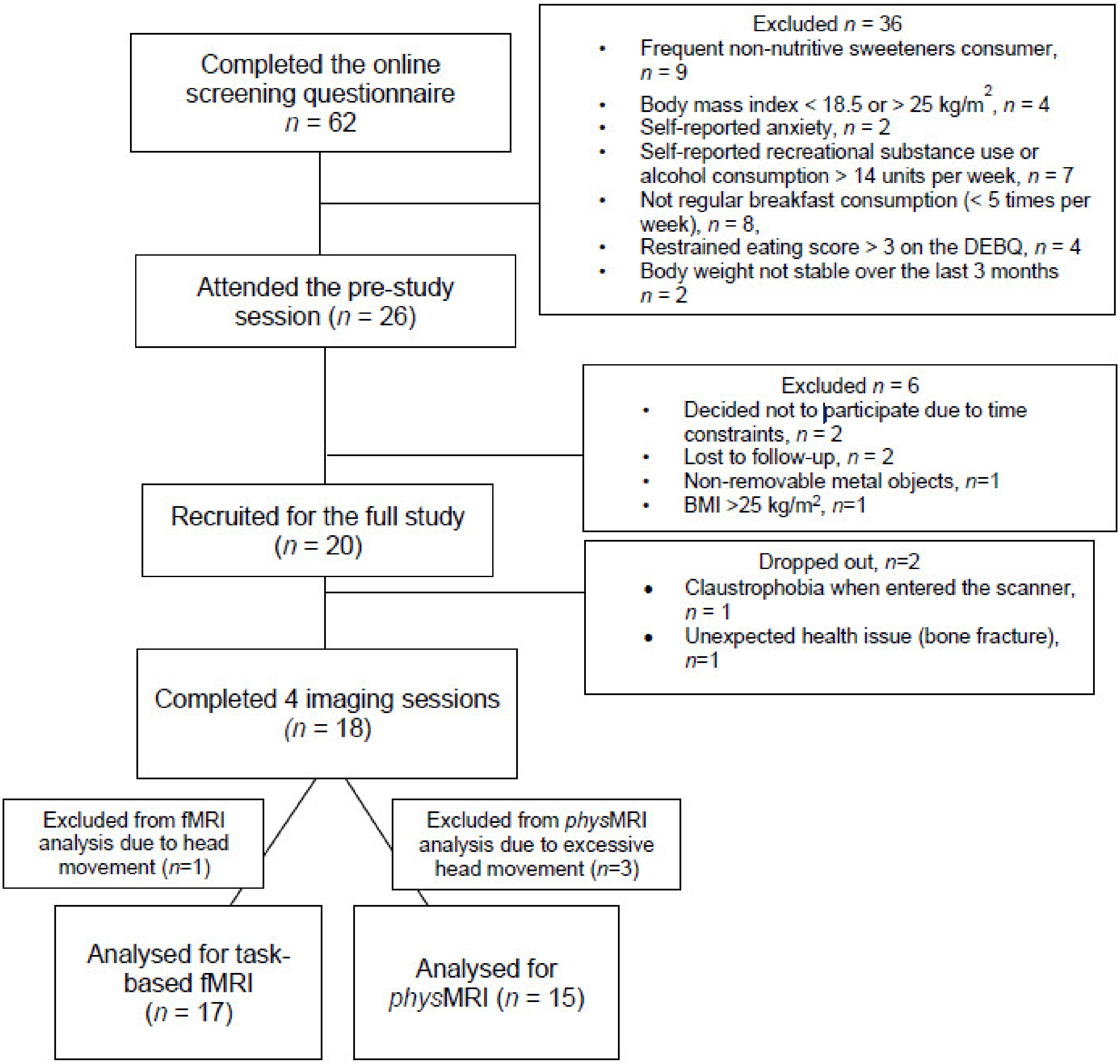

Figure S2. Participant flow chart. fMRI, functional magnetic resonance imaging; *phys*MRI, physiological-MRI; DEBQ, Dutch Eating Behavior Questionnaire; BMI, body mass index.

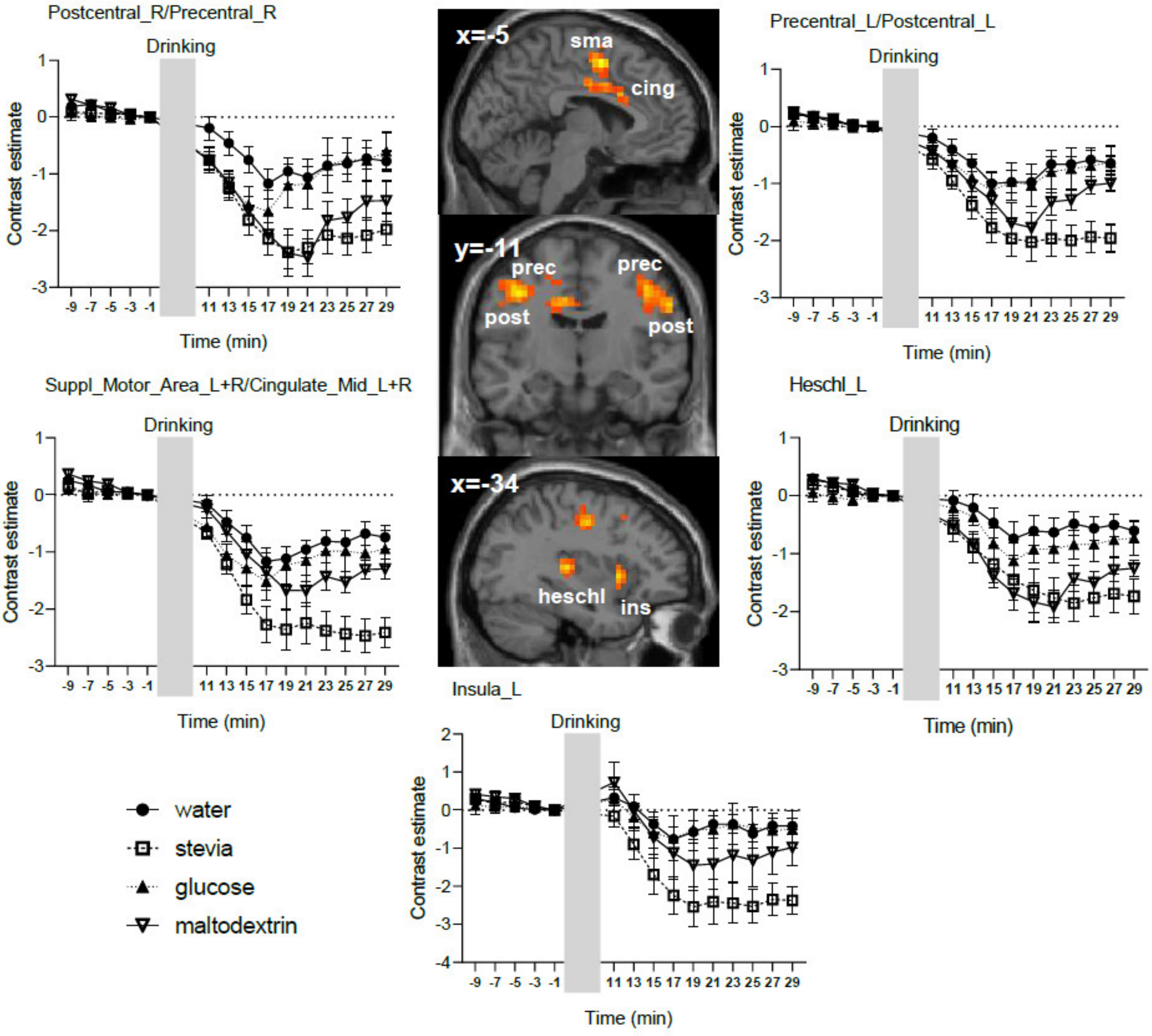

Figure S3. Line graphs present changes in blood-oxygenation-level-dependent (BOLD) signal over time in clusters that showed a significant interaction of taste-by-calories-by-time, *n* = 15. Brain overlays show areas exhibiting significant effect of taste-by-calories-by-time at a cluster extent $P_{FWE} < 0.05$ at a height threshold of $p < 0.001$ uncorrected. Cing, cingulate cortex; heschl, Heschl's gyri or tranverse temporal gyrus; ins, insula; post, postcentral gyrus; prec, precentral gyrus; sma, supplementary motor area.

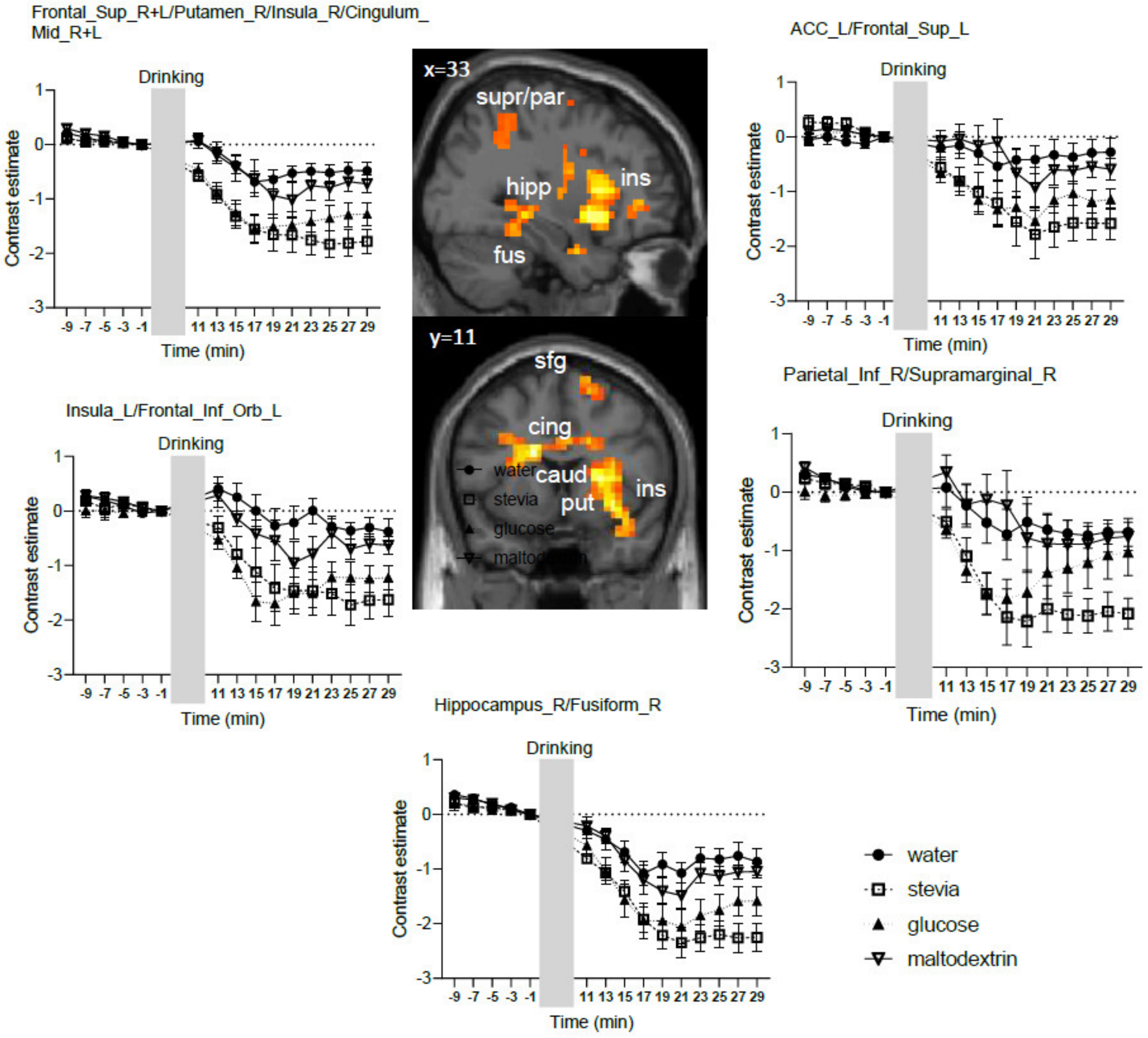

Figure S4. Line graphs present changes in blood-oxygenation-level-dependent (BOLD) signal over time in the significant clusters following oral ingestion of the sweet (stevia, glucose) compared to the non-sweet beverages (water, maltodextrin), n = 15. Brain images show areas exhibiting significant effect of time for sweet versus non-sweet beverages. Results correspond to cluster extent $P_{FWE} < 0.05$ at a height threshold of $p < 0.001$ uncorrected. Caud, caudate; cing, cingulate cortex; fus, fusiform gyrus; hipp, hippocampus; ins, insula; put, putamen; sfg, superior fro ntal gyrus; supr/par, supramarginal gyrus/inferior parietal lobule.

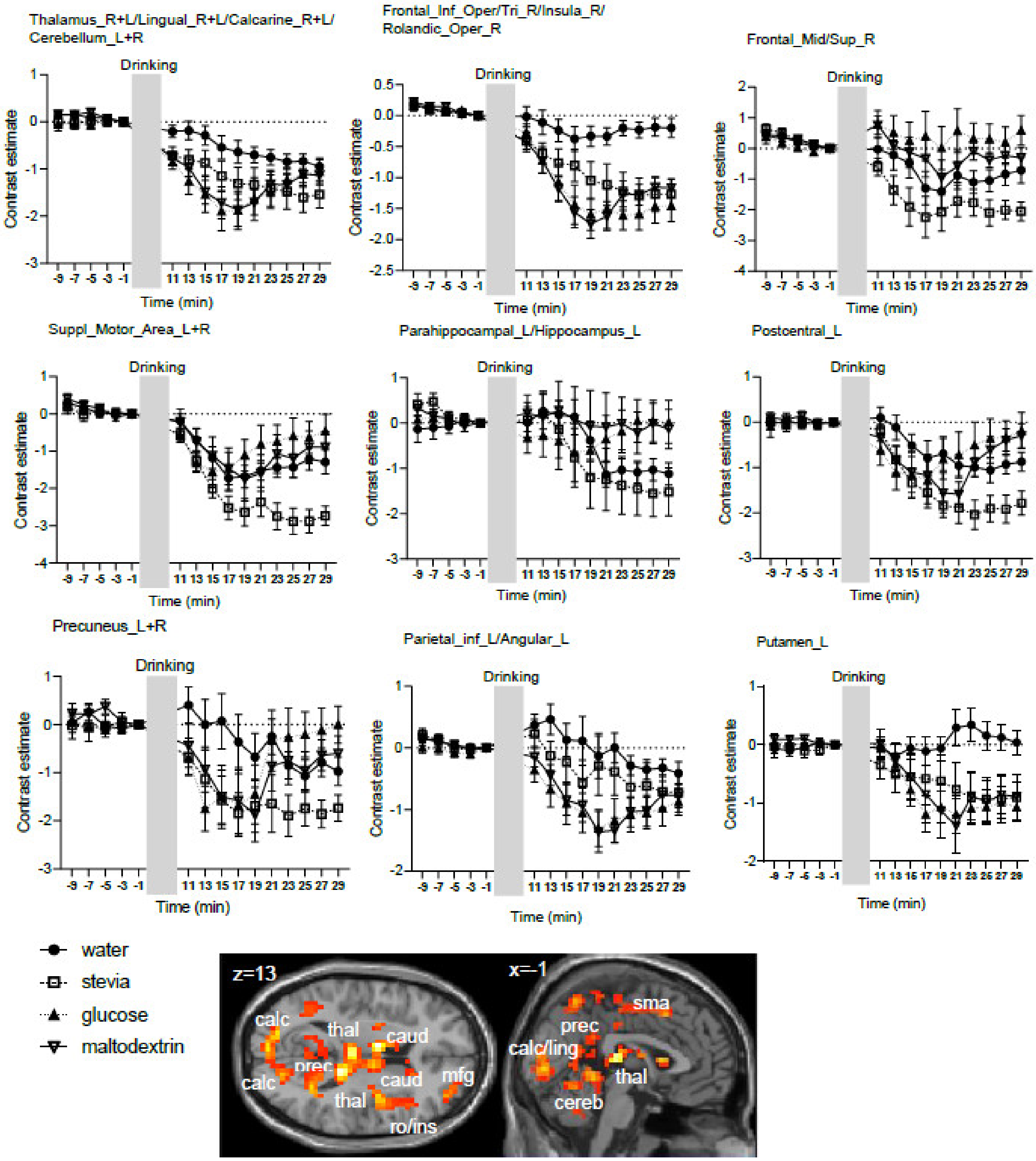

Figure S5. Line graphs present changes in blood-oxygenation-level-dependent (BOLD) signal over time in selected clusters that showed a significant effect of time in the comparison of caloric (glucose, maltodextrin) compared to the non-caloric beverages (water, stevia), $n = 15$. Brain overlays show areas exhibiting significant effect of calories-by-time at cluster extent $P_{FWE} < 0.05$ at a height threshold of $p < 0.001$ uncorrected. Calc, calcarine cortex; caud, caudate; cereb, cerebellum; ling, lingual gyrus; mfg, middle frontal gyrus; prec, precuneus; ro/ins, rolandic operculum/insula; sma, supplementary motor area; thal, thalamus.

Table S1. Participants' mood rating before the start of each imaging session, $n = 18$. p values correspond to repeated-measures ANOVA with beverage type as within-subjects variable.

	**Water**	**Stevia**	**Glucose**	**Maltodextrin**	***p* value**
Clear headed	73.56 ± 3.53	75.50 ± 3.62	74.94 ± 3.68	70.06 ± 4.46	0.384
Energetic	67.44 ± 4.54	70.11 ± 4.21	68.72 ± 4.33	62.94 ± 4.82	0.271
Happy	76.22 ± 2.93	79.56 ± 2.91	76.11 ± 4.41	73.06 ± 3.82	0.235
Tired	39.56 ± 5.01	42.33 ± 5.65	44.00 ± 5.75	45.06 ± 5.37	0.848
Relaxed	69.44 ± 4.25	71.61 ± 3.42	72.83 ± 4.18	69.44 ± 4.25	0.737
